# Strand-Specific Quantitative Reverse Transcription-Polymerase Chain Reaction Assay for Measurement of Arenavirus Genomic and Antigenomic RNAs

**DOI:** 10.1371/journal.pone.0120043

**Published:** 2015-05-15

**Authors:** Kelsey Haist, Christopher Ziegler, Jason Botten

**Affiliations:** 1 Department of Microbiology and Molecular Genetics, University of Vermont, Burlington, Vermont, United States of America; 2 Department of Medicine, Division of Immunobiology, University of Vermont, Burlington, Vermont, United States of America; The Scripps Research Institute, UNITED STATES

## Abstract

Arenaviruses are bi-segmented, single-stranded RNA viruses that cause significant human disease. The manner in which they regulate the replication of their genome is not well-understood. This is partly due to the absence of a highly sensitive assay to measure individual species of arenavirus replicative RNAs. To overcome this obstacle, we designed a quantitative reverse transcription (RT)-PCR assay for selective quantitation of each of the lymphocytic choriomeningitis virus (LCMV) genomic or antigenomic RNAs. During the course of assay design, we identified a nonspecific priming phenomenon whereby, in the absence of an RT primer, cDNAs complementary to each of the LCMV replicative RNA species are generated during RT. We successfully circumvented this nonspecific priming event through the use of biotinylated primers in the RT reaction, which permitted affinity purification of primer-specific cDNAs using streptavidin-coated magnetic beads. As proof of principle, we used the assay to map the dynamics of LCMV replication at acute and persistent time points and to determine the quantities of genomic and antigenomic RNAs that are incorporated into LCMV particles. This assay can be adapted to measure total S or L segment-derived viral RNAs and therefore represents a highly sensitive diagnostic platform to screen for LCMV infection in rodent and human tissue samples and can also be used to quantify virus-cell attachment.

## Introduction

Arenaviruses are single-stranded RNA viruses that are capable of establishing asymptomatic, persistent infections in their normal hosts, typically rodents [1,2]. Several arenaviruses cause significant human disease, including the New World viruses Junin [3], Machupo [4], and Guanarito [5], which cause hemorrhagic fever syndromes in South America [6]. Lassa virus and lymphocytic choriomeningitis virus (LCMV), the target virus for this assay, are both Old World arenaviruses. Lassa virus causes Lassa fever along the western coast of Africa [7] while LCMV, which has a worldwide distribution, is responsible for aseptic meningitis [8]. LCMV is also a potent teratogen [9] as well as a highly lethal pathogen in immunosuppressed individuals [10]. In addition, LCMV is the prototypical virus for the family and is widely used as a model organism to study arenavirus replication and pathogenesis, as well as the adaptive immune response to viral infection [8].

**Fig 1:**
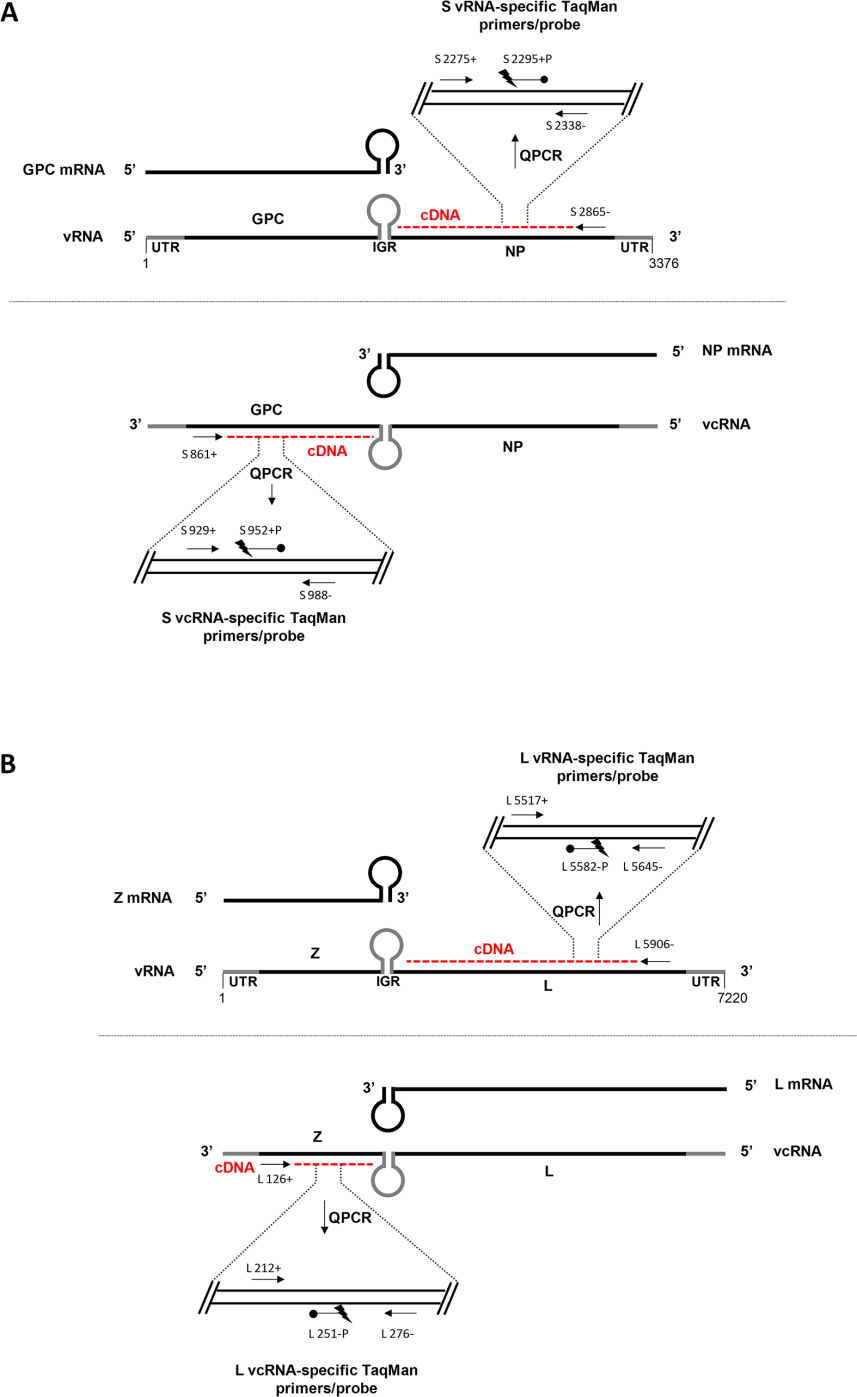
QRT-PCR strategy to enumerate vRNA and vcRNA species. Each of the S and L segment viral RNA species generated during LCMV infection are depicted along with the location of the RT primers and PCR primer-probe sets used to quantitate each unique vRNA or vcRNA (for full primer and probe sequences, see Tables 1 and 3). All primer coordinates reflect the relative position on the genomic vRNA. To determine copy number of an individual vRNA or vcRNA species, an RT reaction is performed with a biotinylated primer that has complementarity only to that particular RNA species (e.g. the primer binds the S vRNA, but not the S vcRNA or S-derived subgenomic mRNAs for GPC or NP). The biotinylated cDNAs generated during the RT reaction are affinity purified using magnetic streptavidin beads and subjected to QPCR using the indicated TaqMan primer-probe set. Absolute copy number is determined through the use of a standard curve that is included in each QPCR run. For illustrative purposes, we have depicted the 3’ termini of viral mRNAs as uniformly containing the entire intergenic region. Please note that there is considerable heterogeneity at the 3’ termini of these transcripts as described in [59]. IGR, intergenic region; UTR, untranslated region.

The arenavirus genome consists of two single-stranded RNA segments, L and S, which each encode two proteins in an ambisense manner [11,12,13]. The L segment encodes the RNA-dependent RNA polymerase (L) and the matrix (Z) proteins, while the S segment encodes the nucleoprotein (NP) and the glycoprotein precursor (GPC) proteins. The L and S genomic RNA segments (vRNAs) are packaged within infectious virus particles [14,15]. Following virion attachment and entry into permissive host cells, the virion-packaged vRNAs, in association with L and NP, are released into the cytoplasm where they serve as templates for both viral genomic replication and transcription (reviewed in [6,15]). This process begins with the transcription of the mRNAs for both NP (on the S segment) and L (on the L segment) from the L and S vRNA templates. After transcription of these mRNAs has progressed to a certain point, the viral polymerase begins replication of the vRNAs to generate antigenomic (vcRNA) or full-length, complementary copies of each vRNA segment. These vcRNAs then serve as templates for the generation of additional L and S segment vRNAs as well as transcription of the mRNAs for both GPC (S segment) and Z (L segment). In total, eight different viral RNA species are generated during arenavirus infection: two vRNAs, two vcRNAs, and four mRNAs (see Fig 1).

LCMV can establish a persistent state of infection in both rodent and *in vitro* cell culture models (reviewed in [8,16,17]). Infectious virus production typically peaks within the first few days of infection, followed by a marked drop in GPC expression as well as release of infectious virus during persistence [18,19]. Infectious virus production, while low during persistence, can also be quite cyclical [20,21,22]. The mechanism(s) by which LCMV regulates the replication and transcription of its genome over the course of infection and the specific details regarding the dynamics of this process are not fully understood. One tool that is needed to address these outstanding questions is a highly sensitive assay to distinguish and quantitate each of the LCMV replicative RNA species. In the current study, we developed a strand-specific, quantitative (Q)RT-PCR assay for quantification of LCMV S and L segment vRNA and vcRNA. We also describe a phenomenon whereby LCMV RNA species are, in the absence of a virus-specific RT primer, nonspecifically primed for cDNA synthesis during RT reactions and a means to circumvent this nonspecific priming for accurate measurement of individual viral RNA species. We used the assay to evaluate LCMV genome replication at acute and persistent time points and also to determine the viral RNA content of purified virions. This assay will enable studies of the dynamics and efficiency of LCMV replication and also provide a highly sensitive diagnostic platform for measurement of total S and L segment-derived viral RNA species.

## Materials and Methods

### Cells, Mice, and Virus

Vero E6 cells (kidney epithelial cells from an African green monkey; CRL-1586, American Type Culture Collection, Manassas, VA) were provided by J. L. Whitton (The Scripps Research Institute, La Jolla) and grown in Dulbecco’s Modified Eagle Medium (DMEM) (11965–118) supplemented with 10% fetal bovine serum, 1% penicillin-streptomycin (15140–163), and 1% HEPES Buffer Solution (15630–130) purchased from Invitrogen (Carlsbad, CA). MC57 cells (a fibrosarcoma cell line derived from a C57BL/6 mouse; CRL-2295, American Type Culture Collection) were provided by M. J. Buchmeier (University of California, Irvine) and were maintained in RPMI 1640 Medium (22400–105, Invitrogen) containing 10% FBS, 1% penicillin-streptomycin, and 1% HEPES Buffer Solution. Both cell lines were grown at 37°C in a humidified incubator containing 5% CO_2_. A working stock of LCMV strain Armstrong 53b was generated in Vero E6 cells, and its infectious titer was determined via plaque assay on Vero E6 cells. The sucrose-banded preparation of LCMV was generated as previously described [23,24] using supernatant collected from Vero E6 cells at 48 hr post-infection (pi).

Infection of Vero E6 or MC57 cells with LCMV was done at a multiplicity of infection (MOI) of 0.1. For the acute time point collections (12, 18 and 48 hr pi) and the 7 d pi time point, cells were not split prior to collection. For the 24 d pi collection, cells were split 1 to 5 at d 10, 14, 17, and 21 pi prior to the collection. For supernatant collections at 48 hr, 7 d, or 24 d pi, the media was changed on infected cells 24 hr prior to collection. Eight week old HLA-A*0201/K^b^ transgenic mice (described in [25]) were given 2 x 10^5^ plaque forming units (PFU) of LCMV strain Armstrong 53b via intraperitoneal inoculation and euthanized on d 4 pi for collection of spleen. The animal experiments were conducted in facilities approved by the Association for Assessment and Accreditation of Laboratory Animal Care at The Scripps Research Institute in La Jolla, CA and all work was approved by The Scripps Research Institute Institutional Animal Care and Use Committee. Every effort was made to minimize suffering.

### RNA Extraction

Viral RNA was extracted from cell-free supernatants or sucrose-banded virions using the QIAamp Viral RNA Mini Kit (52906, Qiagen, Valencia, CA). The RNeasy Mini Kit (74106, Qiagen) was used to extract RNA from infected tissue culture cells or rodent tissues. We followed the manufacturer’s protocol for both RNA extraction methods. Briefly, supernatants were centrifuged for 5 minutes at 1200 RPM to remove any cellular debris prior to RNA extraction. Volumes of 140 μl of supernatant or 15 μl of sucrose-banded virions were subjected to lysis and purified RNA was eluted in 60 μl nuclease-free ddH_2_O. Infected cells from tissue culture were trypsinized at the desired time point pi and counted. Two to 4 million cells were mixed with lysis buffer, homogenized using QIAshredders (79654, Qiagen), and eluted in 50 μl nuclease-free ddH_2_O. Following necropsy at 4 d pi, 10–30 mg of spleen and 600 μl RLT lysis buffer were added to a pre-weighed beadbeater tube (10831, BioSpec Products, Inc., Bartlesville, OK) filled ~1/8 full with 2.5 mm zirconia/silica beads (11079125z, BioSpec Products, Inc). Tissues were homogenized for 30 to 60 seconds in a Mini-Beadbeater-8 (BioSpec Products, Inc.), centrifuged at 14,800 RPM for 3 minutes, and RNA was extracted from the clarified supernatant according to the manufacturer’s protocol. RNA was eluted in 50 μl of nuclease-free ddH_2_O.

### Plasmids and Primers

The pT7-L and pT7-S plasmids used to establish the standard curves for the QRT-PCR assay contain the complete L and S segment, respectively, of LCMV strain Armstrong 53b and were provided by J. C. de la Torre (The Scripps Research Institute, La Jolla) [26]. The RT primers listed in Table 1, the standard PCR primers in Table 2, and the standard PCR primers shown in Figs 4 and 5 [S 1+ (5’-CGCACCGGGGATCCTAGGCTTTTTGG-3’), S1489+ (5’-AGCCACACCGATTAACCAACAAAG-3’), S 1856+ (5’-TACTCCCTCGAAGCTTCCCTGGTC-3’), S 1515- (5’-TTCCTTTGTTGGTTAATCGGTGTG-3’), S 1785- (5’-AAGAAAGAGATCACCCCGCACTGT-3’), and S 3376- (5’-CGCACAGTGGATCCTAGGCATTTGA-3’)] were obtained from Integrated DNA Technologies, Inc. (Coralville, IA). Biotinylated versions of the RT primers were HPLC purified. The TaqMan primers (4304971) and minor grove binder (MGB) probes (4316033) listed in Table 3 were obtained from Life Technologies (Carlsbad, CA). Each MGB probe was labeled with the 5’ reporter dye 6-carboxyfluorescein (FAM) and a 3’ non-fluorescent quencher (NFQ) attached to a minor groove binder (MGB) moiety. Note that during RT primer design we used the software program Amplify to verify that each primer would only be predicted to anneal to the intended target viral RNA species but not other LCMV replicative or transcriptional RNA species.

**Table 1:** RT primers.

Viral RNA target	RT primer	Sequence (5’ to 3’)
S vRNA	S 2865-	CAGGGTGCAAGTGGTGTGGTAAGA
S vcRNA	S 861+	AGGAGACTAGCGGGCACATTC
L vRNA	L 5906-	TGGGACTGAGTTTCGAGCATTACG
L vcRNA	L 126+	ATAGTACAAACAGGGCCGAAATCC

**Table 2:** Standard RT-PCR primer sets.

Viral RNA target	PCR primer	Sequence (5’ to 3’)
S vRNA	S 2275+	CGCTGGCCTGGGTGAAT
	S 2628-	GAACAGCAGTCCAGCATCAAC
S vcRNA	S 929+	TTGCCTGACCAAATGGATGAT
	S 1163-	TCTCAAGTGGTTCCTCATCAGTAG
L vRNA	L 5517+	GGCCTTGTATGGAGTAGCACCTT
	L 5816-	TTGGAGCTCACCCGATAATG
L vcRNA	L 212+	TTGGTAAGATGCCATGACCACTA
	L 356-	CTTCGTAGGGAGGTGGAGAGC

**Table 3:** Primers and probe sets for QRT-PCR.

Viral RNA target	QPCR primers & probe	Sequence (5’ to 3’)
S vRNA	S 2275+	CGCTGGCCTGGGTGAAT
	S 2338-	ATGGGAAAACACAACAATTGATCTC
	S 2295+P^a^	FAM-CTGCAGGTTTCTCGC-MGBNFQ^b^
S vcRNA	S 929+	TTGCCTGACCAAATGGATGAT
	S 988-	ACTGCTGTGTTCCCGAAACAC
	S 952+P	FAM-TTGCTGCAGAGCTTA- MGBNFQ
L vRNA	L 5517+	GGCCTTGTATGGAGTAGCACCTT
	L 5645-	GGTCTGTGAGATATCAAGTGGTAGAATG
	L 5582-P	FAM-CTGAAGAATACCACCTATTATACCA- MGBNFQ
L vcRNA	L 212+	TTGGTAAGATGCCATGACCACTA
	L 276-	TCGGATACTGACAGCAGAAGGTT
	L 251-P	FAM-AACAGTGCCTGCAAAG- MGBNFQ

^a^P, TaqMan probe.

^b^MGBNFQ, minor groove binder and non-fluorescent quencher dye.

### Reverse Transcription (RT)

A single RT protocol was used to generate cDNA prior to either standard or quantitative (Q)PCR. To prime the synthesis of cDNA from the LCMV S vRNA, S vcRNA, L vRNA, or L vcRNA, we used primers S 2865-, S 861+, L 5906-, or L 126+, respectively (see Table 1). To test for primer-independent cDNA synthesis during RT, we also performed reactions without a primer or without both the RT primer and RT enzyme. RT reactions were carried out in a total volume of 50 μl using the TaqMan Reverse Transcription Reagents Kit (4304134, Life Technologies) per the manufacturer’s guidelines. For each reaction, 5 μl of RNA template was used along with 9 pmol RT primer (biotinylated or not) and incubated at 25° C for 10 minutes, 48° C for 30 minutes, and 95° C for 5 minutes. Following RT, cDNAs were either purified using streptavidin-coated magnetic beads as described below or added directly to the PCR reaction.

### Affinity Purification of Biotinylated cDNAs

Biotinylated cDNAs were purified using streptavidin-coated magnetic beads (11205D, Dynabeads M-280 Streptavidin, Life Technologies) and the Dynabeads kilobaseBINDER Kit (60101, Life Technologies). For each RT reaction, a volume of 10 μl of magnetic beads was pre-equilibrated in 40 μl of Binding Solution. Once pre-equilibrated, 20 μl of the RT reaction was added to each aliquot of beads and incubated for 1.5 hr at room temperature on a rotator. Tubes were then placed on a DynaMag-96 Side Magnetic Particle Concentrator (123.31D, Life Technologies) to immobilize the beads in complex with the biotinylated cDNAs and the supernatant was removed. Each tube was then washed 3 times in 1X Washing Buffer and resuspended in the appropriate volume of PCR master mix, as indicated below.

### Standard PCR

Following RT, cDNAs were either added directly to the PCR master mix or affinity purified using streptavidin beads as described above and resuspended in the PCR master mix. Standard PCR was performed using the *Taq* DNA Polymerase kit (18038–042, Life Technologies) as directed by the manufacturer for the “Basic PCR Protocol”. A total reaction volume of 50 μl was used along with a 1 μM final concentration of each primer and 1.5 units of *Taq* DNA Polymerase. For standard RT cDNA templates that were not subjected to affinity purification, 5 μl of the RT reaction was added to the PCR master mix. As shown in Table 2, LCMV Armstrong 53b S segment vRNA was detected with RT primer S 2865- and PCR primers S 2275+ and S 2628-, S vcRNA with RT primer S 861+ and PCR primers S 929+ and S 1163-, L vRNA with RT primer L 5906- and PCR primers L 5517+ and L 5816-, and L vcRNA with RT primer L 126+ and PCR primers L 212+ and L 356-. Alternatively, as shown in Fig 5, LCMV S segment vRNA was also detected with RT primer S 2865- and PCR primers S 1856+ and S 2628-. In Fig 4, the following PCR primer pairs were used to map the cDNAs formed during RT in the absence of an RT primer: S 1+ and S 3376-, S 1+ and S 1515-, S 1+ and S 1785-, S 1489+ and S 3376-, and S 1856+ and S 3376-. Thermocycling conditions were as follows: 94° for 10 minutes, 30 or 40 cycles of 94° for 30 seconds, 47° for 30 seconds, and 72° for 2 minutes 30 seconds, then 72° for 7 minutes for Fig 3; 94° for 10 minutes, 40 cycles of 94° for 30 seconds, 47° for 30 seconds, and 72° for 4 minutes, then 72° for 7 minutes for Fig 4; and 94° for 10 minutes, 40 cycles of 94° for 30 seconds, 47° for 30 seconds, and 72° for 2 minutes 30 seconds, then 72° for 7 minutes for Fig 5.

### QPCR

Following RT, cDNAs were either added directly to the QPCR master mix or affinity purified using streptavidin beads as described above and resuspended in the QPCR master mix. The QRT-PCR was performed using TaqMan Universal PCR Master Mix (4326614, Life Technologies) and custom primer-probe sets. Each QPCR reaction was carried out in a total volume of 25 μl containing 5 μl of cDNA template, 900 nM of each QPCR primer, and 200 nM of probe. As shown in Fig 1 and Tables 1 and 3, LCMV Armstrong 53b S segment vRNA was detected using RT primer S 2865- and PCR primers-probe S 2275+, S 2338-, and S 2295+P, S vcRNA with RT primer S 861+ and PCR primers-probe S 929+, S 988-, and S952+P, L vRNA with RT primer L 5906- and PCR primers-probe L 5517+, L 5645-, and L 5582-P, and L vcRNA with RT primer L 126+ and PCR primers L 212+, L 276-, and L251-P. Reaction conditions were 95°C for 10 min and 40 cycles of 95°C for 15 seconds and 60°C for 1 min. Absolute copy numbers of vRNA or vcRNA corresponding to the LCMV Armstrong 53b S or L segment were determined by comparison with a series of standard quantities (50, 5x10^3^, 5x10^5^, or 5x10^7^ copies) of the pT7-S or pT7-S plasmids, respectively. Data was acquired using Applied Biosystems 7300 or StepOnePlus Real-Time PCR Systems and analyzed with the provided software.

## Results

### Strand-Specific QRT-PCR Assay Design

To develop an assay that could be used to quantitate each of the replicative viral RNA species generated during LCMV infection, we designed RT primers specific to a unique region of each particular RNA species (vRNA or vcRNA for both the L and S segment) (Fig 1 and Table 1). We reasoned that selective targeting of the individual viral RNA species in the RT step would permit us to enumerate each of these different RNA species in subsequent QPCR reactions. RT primers S 2865- and L 5906- bind to the NP region or the L region, respectively, of the vRNAs and will only prime cDNA synthesis of the vRNA. RT primers S 861+ and L 126+ bind the GPC region or Z region, respectively, of the corresponding vcRNAs and prime cDNA synthesis of only these vcRNA species.

The TaqMan QPCR featured two sets of primers and probe for each segment: for the S segment, one set was specific for the NP region and the other for the GPC region; for the L segment, one set was specific for the L region and the other for the Z region. For the S segment, vRNAs primed by RT primer S 2865- would undergo QPCR using the NP-specific primer-probe set (primers S 2275+/S 2338- and probe S 2295+P) to determine copies of S segment vRNA whereas vcRNAs primed by RT S 861+ would be enumerated using the GPC primer-probe set (primers S 929+/S 988- and probe S 952+P) (Fig 1A and Table 3). For the L segment, vRNAs primed with RT primer L 5906- would be quantified using the L-specific primer-probe set (primers L 5517+/L 5645- and probe L 5582-P) while vcRNAs primed with RT primer L 126+ would be screened with the Z-specific primer-probe set (primers L 212+/L 276- and probe L 251-P) (Fig 1B and Table 3).

**Fig 2:**
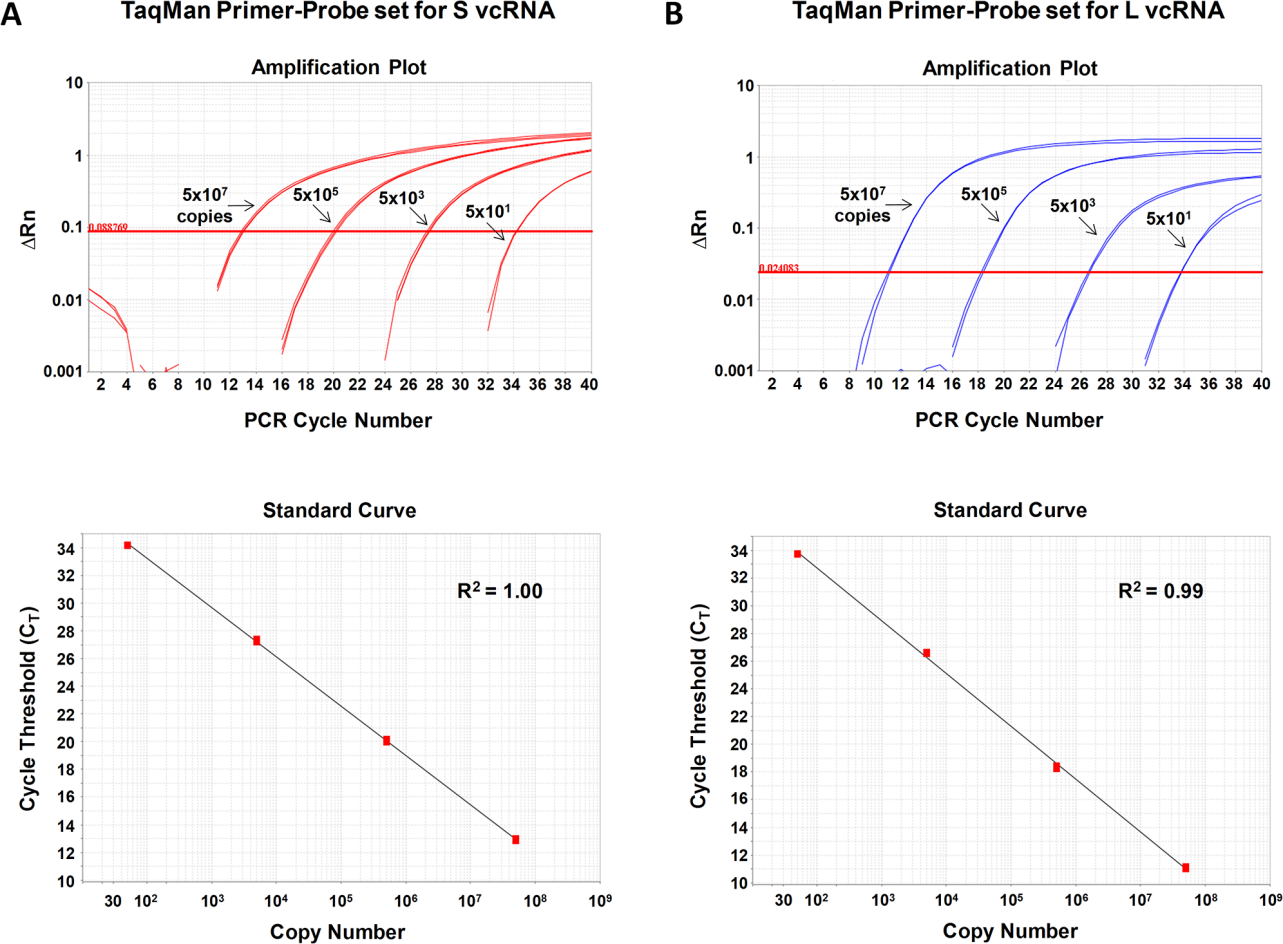
Validation of TaqMan primer-probe sets. Each L or S segment-specific TaqMan primer-probe set was tested for its ability to accurately measure known quantities (50, 5x10^3^, 5x10^5^, or 5x10^7^ copies) of a DNA standard control plasmid encoding the target sequence. Depicted are the amplification plots and standard curve fit lines for the primer-probe sets specific for S segment vcRNA (A) or L segment vcRNA (B).

To determine the absolute copy number of each viral RNA species, we established a standard curve using known copies of a DNA plasmid containing the full-length LCMV S or L segment, respectively [26]. We were routinely able to generate standard curves over a large dynamic range (50 to 5 x 10^9^ copies) with correlation coefficient (R^2^) values of 0.995 or better for each primer-probe set (Fig 2 and data not shown).

### Nonspecific Conversion of Arenavirus RNA Species into cDNA during RT

**Fig 3:**
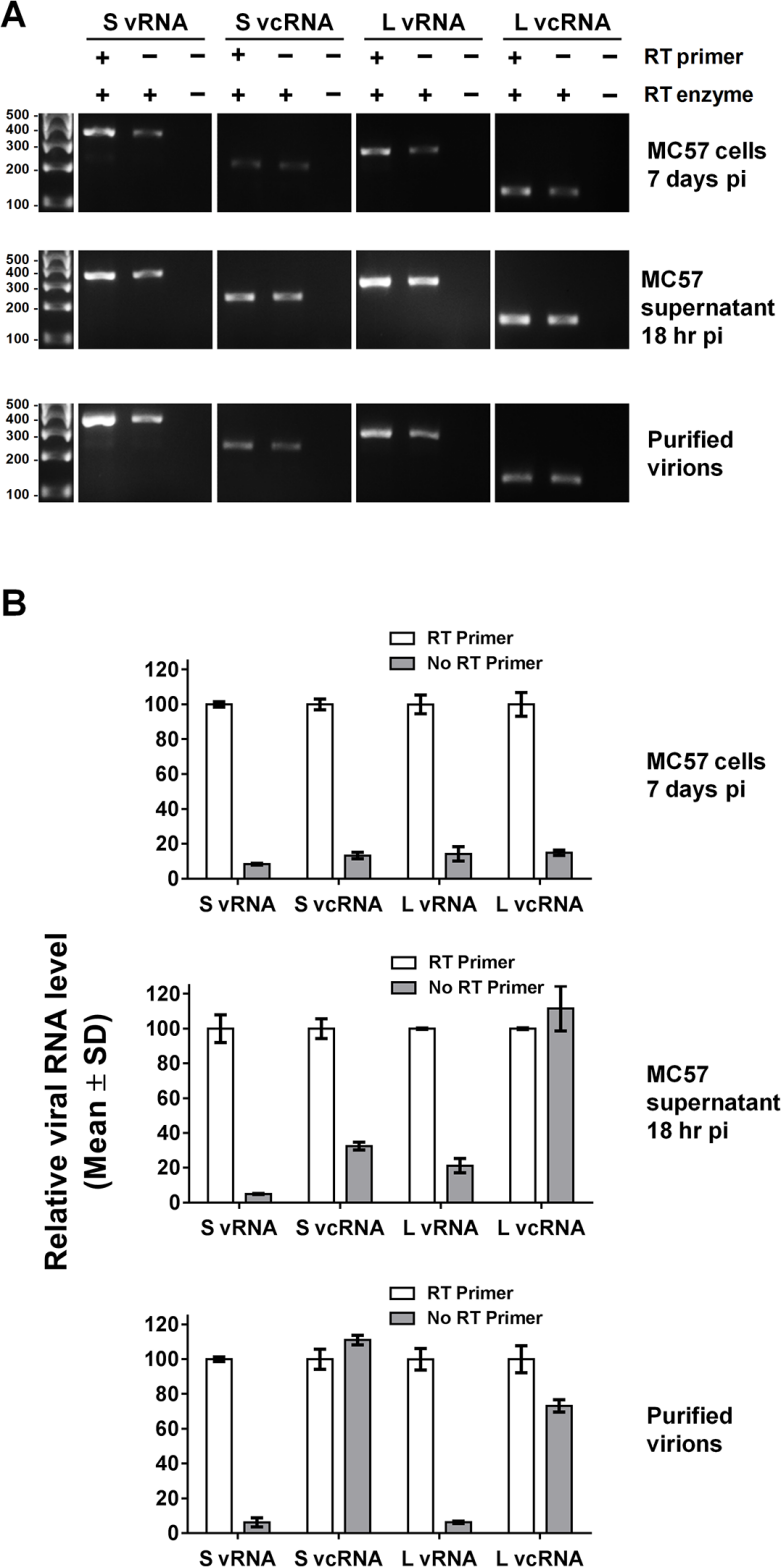
Nonspecific conversion of arenavirus RNA species into cDNA during RT. (A) RNA was extracted from i) MC57 cells 7 d after infection with LCMV, ii) cell-free supernatant collected from MC57 cells 18 hr pi with LCMV, or iii) sucrose-banded LCMV particles collected from Vero E6 cells at 48 hr pi. Each sample was subjected to standard RT-PCR targeting the vRNAs or vcRNAs of the L or S segment, as indicated, using the primers described in Tables 1 and 2. In each case, the RT was performed with: i) the RT enzyme and an RT primer, ii) the RT enzyme but no RT primer, or iii) no RT enzyme and no RT primer. It should be noted that the data shown in panel (A) were obtained during the linear and/or plateau phase of the PCR reaction. Therefore, these data are not suitable for exact quantitation, but rather reflect the presence or absence of a particular cDNA. (B). RNA extracted from the samples listed in panel (A) of this figure were subjected to QRT-PCR targeting the vRNAs or vcRNAs of the L or S segment, as indicated, using the primers and probes described in Tables 1 and 3. Similar to panel (A), in each case, the RT was performed with: i) the RT enzyme and an RT primer, ii) the RT enzyme but no RT primer, or iii) no RT enzyme and no RT primer. For each particular viral RNA species, data are presented as mean ± SD relative to the value obtained using the RT primer.

The assay, as described above, should have been sufficient to accurately enumerate the replicative RNA species generated during LCMV infection. However, when RT reactions were performed in the absence of an RT primer as a negative control, a cDNA product was amplified by each of the L and S segment primer-probe sets in a subsequent PCR (Fig 3). This occurred for viral RNA extracted from LCMV-infected cells, supernatants derived from LCMV-infected cells, sucrose-banded virions, and LCMV-infected rodent tissues (Fig 3 and Table 4). The quantity of cDNA detected in the absence of an RT primer varied depending on the source of viral RNA and the particular RNA species targeted. In the case of LCMV-infected cells harvested over a range of acute and persistent time points, the quantity of cDNA generated in the absence of an RT primer ranged from 6.7 ± 0.2 (mean ± SD) to 29.3 ± 0.9% of the signal obtained using an RT primer specific for a particular RNA species (Fig 3B and Table 4). For viral RNA extracted from the spleens of mice 4 days following LCMV infection, the signal generated in the absence of an RT primer was as high as 6.8 ± 0.4% of that seen with the specific RT primer for the L segment vRNA and S segment vRNA and vcRNA whereas the values ranged from 44.8 ± 6.6 to 58.4 ± 3.0% for L segment vcRNA. For cell-free or sucrose-banded virions, 4.0 ± 0.7 to 14.7 ± 0.3% of the primer-specific signal was detected in the absence of an RT primer for the L and S segment vRNAs. However, values for L and S segment vcRNAs in the absence of an RT primer sometimes equaled or slightly exceeded that seen with the specific RT primer.

**Table 4:** Percent of primer-specific target RNA signal generated in the absence of an RT primer.

Source of RNA	Time pi	Percent of target RNA^a^			
		S segment		L segment	
MC57 cells		**vRNA**	**vcRNA**	**vRNA**	**vcRNA**
	12 hr	nd^b^	nd	18.2 ± 0.5	23.1 ± 0.7
	18 hr	7.4 ± 0.3	14.5 ± 0.1	16.1 ± 1.0	29.3 ± 0.9
	7 d	8.1 ± 0.8	15.1 ± 0.8	18.9 ± 0.7	17.1 ± 0.1
	7 d	8.8 ± 0.1	11.2 ± 0.1	14.3 ± 0.6	14.0 ± 0.1
	7 d	8.4 ± 0.1	13.7 ± 0.6	9.7 ± 0.1	14.1 ± 0.2
	14 d	9.7 ± 3.8	11.9 ± 0.1	nd	nd
	24 d	6.7 ± 0.2	14.0 ± 0.5	16.0 ± 1.1	11.2 ± 0.3
Mouse spleen #1	4 d	5.4 ± 4.9	6.8 ± 0.4	3.4 ± 0.3	44.8 ± 6.6
Mouse spleen #2	4 d	3.9 ± 0.8	5.3 ± 2.0	2.2 ± 2.3	58.4 ± 3.0
Supernatant from MC57 cells	12 hr	4.0 ± 0.7	8.7 ± 0.9	14.7 ± 0.3	102.6 ± 11.4
	18 hr	5.0 ± 0.3	32.5 ± 2.2	21.2 ± 4.1	111.7 ± 12.9
	24 d	6.9 ± 3.7	5.2 ± 1.4	nd	nd
Sucrose-banded virions from Vero E6 cells	48 hr	6.2 ± 2.6	111 ± 2.8	6.3 ± 0.7	73.1 ± 3.6

^a^For each RNA species, the numerical values represent the mean copy number ± standard deviation obtained via QRT-PCR in the absence of an LCMV segment-specific primer during the RT step divided by the copy number obtained when the segment-specific primer was used during RT. Each value was multiplied by 100 to convert to percentage.

^b^nd, not done.

To determine whether the template being amplified in the PCR reaction was a DNA intermediate of the LCMV genome [27], we conducted PCR directly on RNA samples in the absence of an RT step. No amplification was detected indicating that the template is an RNA molecule that is converted to cDNA during the RT step (Fig 3A).

In summary, the LCMV L and S segment RNA species are susceptible to a nonspecific cDNA priming phenomenon during RT reactions. It should be noted that this is not unique to LCMV as we observed a similar nonspecific priming event using RNA extracted from cell-free Junin virus Candid#1 virions (data not shown).

### Mapping the Nonspecifically-Primed cDNAs

**Fig 4:**
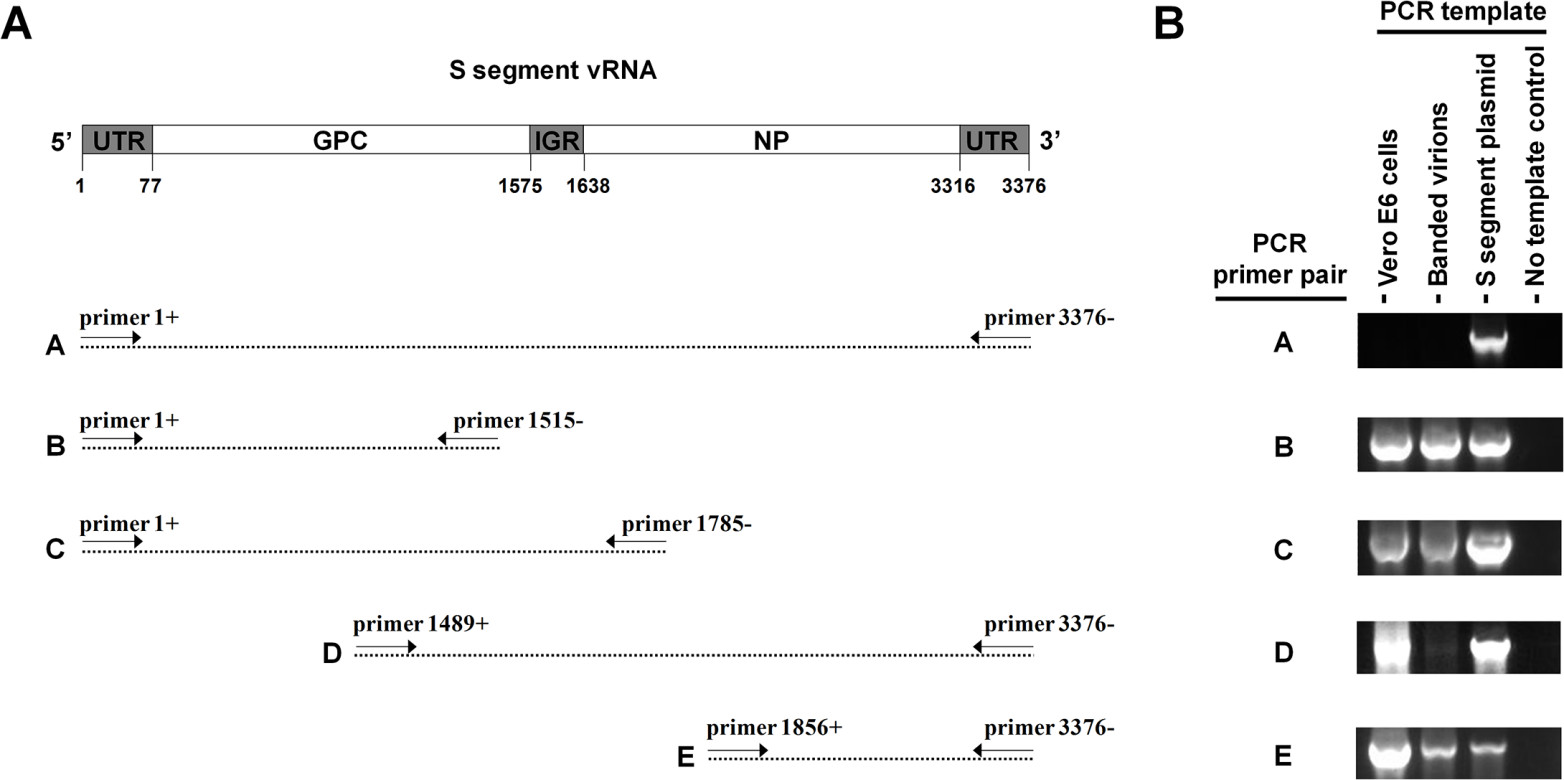
Mapping the nonspecifically-primed cDNA. RNA extracted from i) Vero E6 cells 48 hr pi with LCMV or ii) sucrose-banded LCMV particles collected from Vero E6 cells at 48 hr pi was subjected to RT in the absence of an RT primer. To determine the extent of nonspecific cDNA conversion of the LCMV S segment, the resulting cDNA products were screened via standard PCR using a panel of PCR primer pairs. Panel (A) shows a depiction of the LCMV S segment vRNA and the region of the segment covered by each PCR primer pair. Primer coordinates reflect the relative position on the S segment vRNA. Panel (B) shows the results of the PCR for each primer pair. Controls included the pT7 S segment plasmid that encodes the intact LCMV S segment as well as a no template control.

To map the nonspecifically-primed cDNAs, viral RNA derived from either Vero E6 cells at 48 hr pi or sucrose-banded virions was subjected to RT without an RT primer. The resulting cDNAs were then used in a series of PCR reactions to determine the region of the LCMV S segment that was converted to cDNA (Fig 4). While we were unable to amplify a full-length (3376 bp) S segment PCR product, we did detect products covering large portions of the S segment. A 1785 bp PCR product was detected from both RNA samples using primers covering the S segment from its 5’ terminus (position 1 relative to the vRNA) through both the adjacent GP gene and the IGR to position 1785, which lies in the NP gene just beyond the IGR (see primer pair C in Fig 4). Inversely, an 1887 bp product ranging from the 3’ terminus (position 3376 relative to the vRNA) through both the adjacent NP gene and the IGR to position 1489 in the GPC gene was amplified from both samples, although the signal from the virion RNA preparation was very faint compared to the Vero E6 cell-derived RNA sample (see primer set D in Fig 4). We also amplified a slightly smaller (1501 bp) PCR product from both samples that ranged from the 3’ terminus (position 3376 relative to the vRNA) to the far side of the NP gene, just prior to the IGR (position 1856 relative to the vRNA) (see primer set E in Fig 4). In summary, our data suggest that large regions of the S segment are converted to cDNA in a primer-independent manner during RT. While our data indicate that the extent of cDNA conversion is similar for viral RNA from cells versus virions, the efficiency of this conversion appears to be greater in host cells.

### Selective Affinity Purification of cDNAs Primed with Biotinylated RT Primers Circumvents Nonspecific Priming

**Fig 5:**
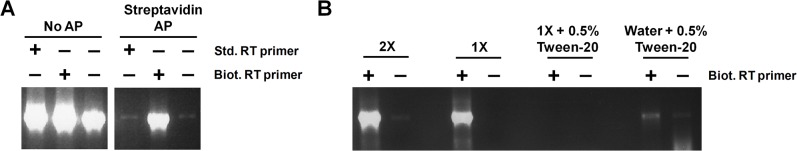
Affinity purification of cDNAs primed with biotinylated RT primers circumvents nonspecific priming during RT. (A and B) RNA extracted from a high titer stock of cell-free LCMV virions was subjected to standard RT-PCR to detect S segment vRNA using the RT primer S 2865- and PCR primers 1856+ and 2628- (note that the sequence for primer 1856+ is listed in the Methods). In panel (A), three RT conditions were tested. The first RT condition featured a standard RT primer, the second had a biotinylated primer, and the third had no RT primer, as indicated. A portion of each reaction was subjected to affinity purification using streptavidin magnetic beads and then both the input and streptavidin-purified cDNAs were subjected to PCR. In panel (B), two RT conditions were tested: one with a biotinylated RT primer and the other without an RT primer. In an attempt to eliminate nonbiotinylated cDNAs from nonspecifically binding to streptavidin beads, a panel of four wash buffers (the 2X wash buffer from the Dynabeads kilobaseBINDER Kit, a 1X dilution of this buffer alone or containing 0.5% Tween 20, or water containing 0.5% Tween 20) were used during affinity purification. Following affinity purification, the captured cDNAs were subjected to PCR.

Nonspecific priming of viral RNAs during RT has been reported for several RNA viruses [28,29,30,31,32,33,34,35]. One effective strategy to circumvent this phenomenon has been the use of biotinylated RT primers to generate target-specific cDNAs and streptavidin-coated magnetic beads to affinity purify these biotinylated cDNAs for downstream quantitation via QPCR [29,30,36,37]. To test whether this strategy would work in our system, we performed traditional RT-PCR on an RNA sample derived from a high titer stock of cell-free LCMV virions. Three RT conditions were tested: one with a standard RT primer, one with a biotinylated RT primer, and one without an RT primer. Following RT, a fraction of each sample was subjected to affinity purification with streptavidin-coated beads as described in the Methods and then both input and affinity purified cDNAs were subjected to PCR. As expected, a positive PCR signal was detected for each of the 3 unpurified cDNA samples (see Fig 5A). For the samples subjected to affinity purification, a strong signal was detected from the sample containing the biotinylated RT primer while a faint signal was detected in the other two conditions, indicating elimination of most of the undesired non-specific product, but not all.

We next tested whether increasing the stringency of the wash step in the affinity purification protocol would eliminate carry-over of residual non-biotinylated cDNA. A panel of wash buffers was tested including the 2X Washing Buffer supplied with the Dynabeads kilobaseBINDER kit (used in Fig 5A), 1X Washing Buffer, 1X Washing Buffer containing 0.5% Tween-20, or water containing 0.5% Tween-20. The only wash buffer that eliminated the non-specific product without affecting the retention of the biotinylated product was the 1X Washing Buffer (Fig 5B). We validated that the protocol was effective at removing all nonspecific template for each of the remaining 3 RT primers (data not shown). Based on these results, we elected to exclusively use the 1X Washing Buffer in all subsequent experiments.

### Assay Optimization

**Fig 6:**
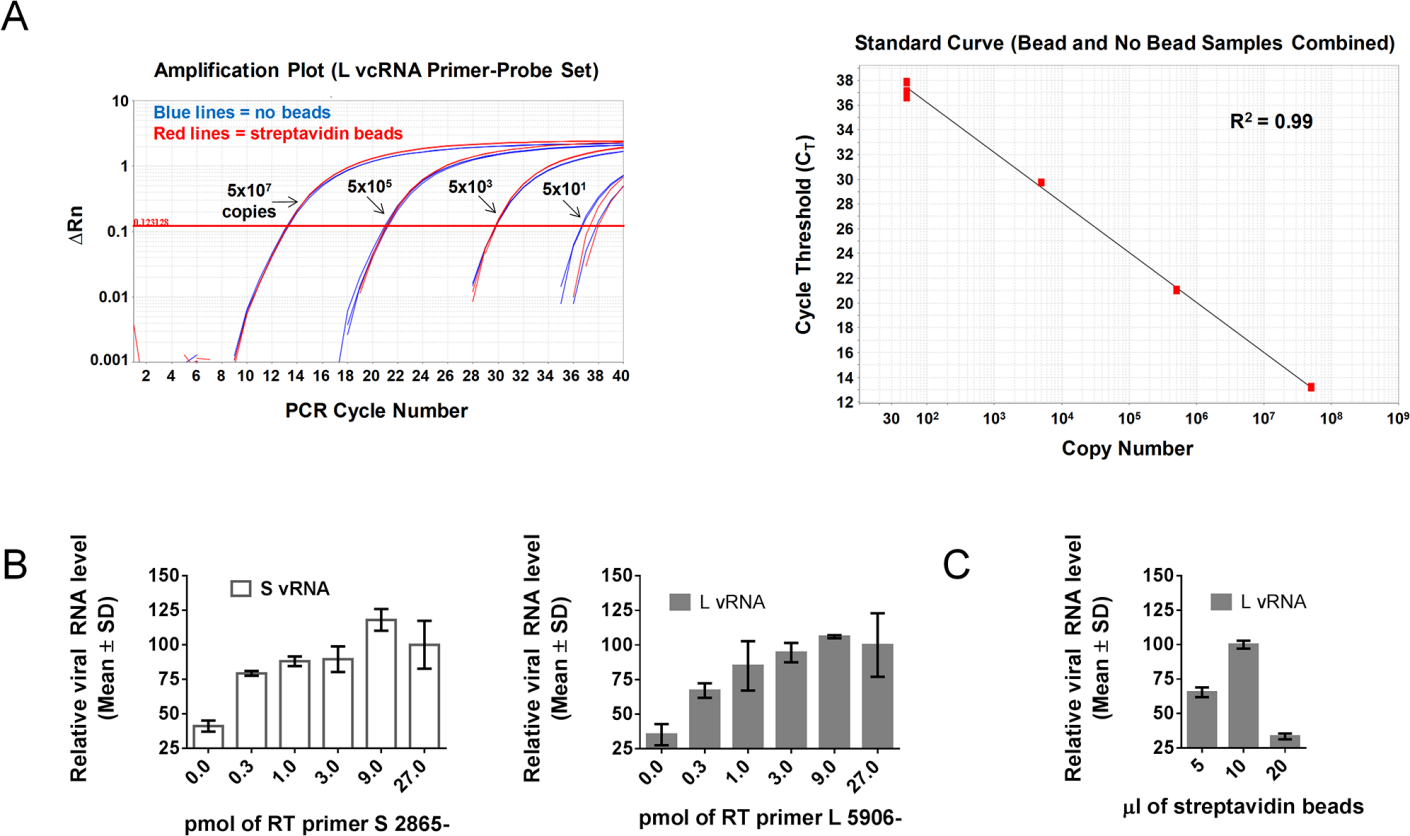
Optimization of assay conditions. (A) Streptavidin beads do not impact QPCR efficiency. QPCR featuring the primer-probe set specific for LCMV L segment vcRNA listed in Table 3 was conducted using the pT7 plasmid encoding the LCMV L segment. The primer-probe set was tested on a range of plasmid template quantities (50, 5x10^3^, 5x10^5^, or 5x10^7^ copies) with either 25 μl of streptavidin beads (red lines in the amplification plot) or no beads (blue lines in the amplification plot) included in the QPCR reaction mixture. The standard curve fit line was generated by pooling all of the values from samples containing beads or not. Although not shown, similar results were obtained for the remaining primer-probe sets specific for S vRNA and vcRNA or L vRNA. (B) 9 pmol of primer is sufficient to prime total viral RNA template for cDNA synthesis during RT. RNA extracted from sucrose-banded LCMV virions collected from Vero E6 cells at 48 hr pi was subjected to RT using 27, 9, 3, 0.3, or 0 pmol of biotinylated primer S 2865- (to target S vRNA) or L 5906- (to target L vRNA), followed by QPCR using the primer-probe sets for S vRNA or L vRNA, respectively, that are listed in Table 3. Data are presented as mean ± SD relative to the 9 pmol samples. (C) 10 μl of streptavidin beads is sufficient to capture 9 pmol of biotinylated RT primer. RNA extracted from sucrose-banded LCMV particles collected from Vero E6 cells at 48 hr pi was subjected to RT using 9 pmol of biotinylated RT primer S 5906- (specific for L vRNA). Following RT, biotinylated cDNAs were affinity purified using 20, 10, or 5 μl of magnetic streptavidin beads and subjected to QPCR using the primer-probe set for L vRNA listed in Table 3. Data are presented as mean ± SD relative to the 10 μl bead samples.

Our next goal was to optimize the remaining parameters of the assay to maximize its sensitivity while also streamlining the time required to perform it. We first addressed whether biotinylated cDNAs captured on streptavidin beads needed to be eluted from the beads prior to PCR or alternatively whether they could be added directly into the PCR reaction in complex with the beads. Using standard curve plasmids as templates in QPCR reactions, we determined that the addition of up to 25 μl of streptavidin beads into a QPCR reaction had no effect on PCR efficiency for any of the four TaqMan primer-probe sets (Fig 6A and data not shown). This result indicates that an elution step is not necessary to maintain optimal PCR efficiency.

We next determined the concentration of RT primer required to prime the entire pool of target RNA in a given RT reaction. QRT-PCR was performed on RNA extracted from sucrose-banded LCMV particles, as this sample represents the greatest abundance of RNA for which this assay would likely be used. From this experiment, it was determined that 9 pmol of primer was sufficient to fully prime the target RNA template for each of the 4 primers (Fig 6B and data not shown).

To determine the amount of beads required to capture all of the biotinylated cDNAs generated using 9 pmol of RT primer, QRT-PCR was performed on RNA extracted from sucrose-banded LCMV particles. Following RT, biotinylated cDNAs were affinity purified using a range of streptavidin-coated magnetic beads (5 μl, 10 μl, or 20 μl). From this experiment, it was determined that 10 μl of beads was optimal to capture all of the biotinylated cDNAs (Fig 6C).

### Validation of Optimized QRT-PCR Protocol

**Fig 7:**
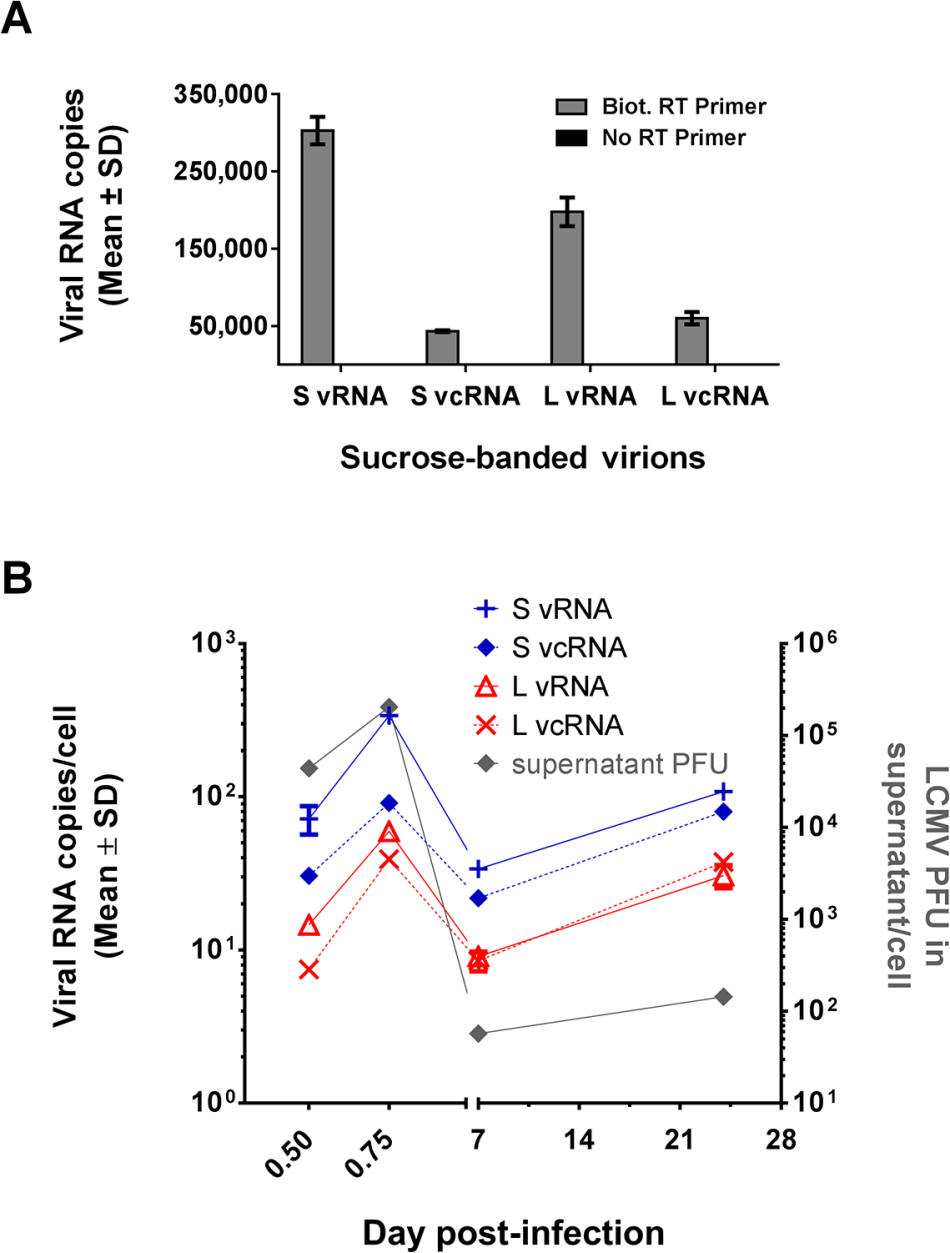
Use of optimized QRT-PCR assay to measure the RNA content in LCMV virions and the dynamics of genome replication during acute and persistent LCMV infection. (A) Viral RNA content of LCMV virions. RNA extracted from sucrose-banded LCMV particles collected from Vero E6 cells at 48 hr pi was subjected to RT using 9 pmol of the biotinylated RT primers listed in Table 1 (gray bars). As a control, RT was also carried out in the absence of an RT primer (black bars). Biotinylated cDNAs were affinity purified using 10 μl of magnetic streptavidin beads and subjected to QPCR using the primer-probe sets for the vRNAs or vcRNAs of the L or S segment that are listed in Table 3. Data are presented as mean ± SD. (B) Dynamics of LCMV replication during acute and persistent LCMV infection. Viral RNA extracted from MC57 cells at 12 h, 18 hr, 7 d, or 24 d pi with LCMV was subjected to RT using 9 pmol of the biotinylated RT primers listed in Table 1. Biotinylated cDNAs were affinity purified using 10 μl of magnetic streptavidin beads and subjected to QPCR using the primer-probe sets for S vRNA or L vRNA, respectively, that are listed in Table 3. Values are listed as mean copies per cell ± SD and are plotted on the left Y axis. For each sample, PFUs in the supernatants were determined by plaque assay and are reported as PFU/cell on the right Y axis.

We tested the optimized quantitative RT-PCR protocol on RNA extracted from sucrose-banded virions (Fig 7A). For each primer-probe set, there was no carryover of nonspecific cDNA from matching control samples that did not receive an RT primer. Importantly, we were able to clearly enumerate the copy number of each species of replicative viral RNA. These results demonstrate that the assay is effective for measurement of replicative RNA species and overcomes the nonspecific cDNA priming phenomenon observed in the LCMV system.

### RNA Content of LCMV Virions

We chose to include the sucrose-banded virions in our screening to determine the viral RNA content of LCMV particles. The mean ratio of S vRNA to L vRNA in the virions was 1.5 ± 0.1 (mean ± SD) (Fig 7A and Table 5). Notably, we were able to detect both L and S segment vcRNA species in the purified virions. The vcRNA species were less abundant than the corresponding vRNA species (7.0 ± 0.3-fold less S vcRNA versus S vRNA; 3.3 ± 0.4-fold less L vcRNA versus L vRNA). The overall level of S vcRNAs was slightly less than L vcRNAs (ratio of 0.7 ± 0.1 S vcRNA to L vcRNA).

**Table 5:** Ratio of viral RNA species found in virions and infected cells.

Source of RNA	Time pi	Ratio of viral RNA species^a^			
		S vRNA to L vRNA	S vRNA to S vcRNA	L vRNA to L vcRNA	S vcRNA to L vcRNA
MC57 cells	12 hr	4.9 ± 1.1	2.3 ± 0.4	2.0 ± 0.1	4.1 ± 0.2
	18 hr	5.7 ± 0.8	3.7 ± 0.5	1.5 ± 0.1	2.3 ± 0.2
	7 d	3.7 ± 0.4	1.6 ± 0.1	1.1 ± 0.2	2.6 ± 0.3
	24 d	3.6 ± 0.7	1.4 ± 0.1	0.8 ± 0.1	2.1 ± 0.1
Sucrose-banded virionsfrom Vero E6 cells	48 hr	1.5 ± 0.1	7.0 ± 0.3	3.3 ± 0.4	0.7 ± 0.1

^a^The ratio of the two viral RNA species indicated at the top of each column was calculated by dividing the value of the first RNA species listed by the value of the second. Ratios are reported as mean ± standard deviation.

### Dynamics of LCMV Replication during Acute and Persistent Infection

We used the optimized assay to investigate the dynamics of LCMV replication at selected time points following infection (12 hr, 18 hr, 7 d, and 24 d). There was a consistent hierarchy in the quantities of viral RNA species observed (S vRNA > S vcRNA > L vRNA > L vcRNA) at 12 hr, 18 hr, and 7 d pi (Fig 7B and Table 5). At 24 d pi, L vcRNA slightly exceeded L vRNA levels and the overall profile was S vRNA > S vcRNA > L vcRNA > L vRNA. As outlined in Table 5, the ratio of S vRNA to L vRNA ranged from 3.7 ± 0.4 (mean ± SD) to 5.7 ± 0.8-fold, which is consistent with previous findings [15]. The ratio of vRNA to vcRNA for either the S or L segment was variable, ranging from 1.4 ± 0.1 to 3.7 ± 0.5 for the S segment and 0.8 ± 0.1 to 2.0 ± 0.1 for the L Segment. Genomic replication over this time frame was cyclical with quantities of each replicative RNA i) increasing from 12 hr to 18 hr pi, ii) decreasing from 18 hr to 7 d pi, and iii) increasing over the final time period from 7 d to 24 d pi (Fig 7B and Table 6). Interestingly, despite a 1424-fold reduction in the quantity of infectious virus released into the supernatant at 18 hr versus 24 d pi, the levels of replicative RNAs decreased by no more than 3.1-fold (3.1 ± 0.3-fold, S vRNA; 1.1 ± 0.1-fold, S vcRNA; 2.0 ± 0.3-fold, L vRNA; and 1.0 ± 0.1-fold, L vcRNA) (Fig 7B). The observed cyclical pattern of genome replication and infectious virus production as well as the inverse relationship between viral RNA and infectious virus during acute versus persistent infection is consistent with previous reports from *in vitro* and *in vivo* models [20,21,38,39]. In summary, these experiments further characterize the dynamics of LCMV replication and highlight the utility of this assay for measuring arenavirus genomic replication.

**Table 6:** Fold-change in LCMV replicative RNA species over the course of infection.

Source of RNA	Times pi compared	Fold-change in viral RNA copy number^a^			
		S vRNA	S vcRNA	L vRNA	L vcRNA
MC57 cells	12 hr to 18 hr	4.9 ± 1.3	3.0 ± 0.1	4.1 ± 0.3	5.2 ± 0.2
	18 hr to 7 d	-10.0 ± 0.8	-4.2 ± 0.1	-6.6 ± 0.6	-4.7 ± 0.6
	7 d to 24 d	3.2 ± 0.2	3.7 ± 0.2	3.4 ± 0.8	4.4 ± 0.3

^a^For each time point comparison, the fold-change was determined by dividing the quantity of a given viral RNA obtained at the earlier time point from the quantity at the later time point. If the resulting value was less than 1 the negative reciprocal is listed (e.g. a value of 0.1 is reported as -10.0). Values are reported as mean ± standard deviation.

## Discussion

Over the course of its replicative cycle within host cells, LCMV generates a total of four replicative RNA species. We describe the development of a highly sensitive QRT-PCR assay to enumerate each of these replicative RNAs. While nonspecific priming of viral RNA into cDNA during RT has been described for other ssRNA viruses [28,29,30,31,32,33,34,35], we provide here the first description of this phenomenon for the arenaviruses, as well as a means to circumvent it, enabling accurate quantitation of copy number after QPCR. Furthermore, our study provides a panel of reagents for measurement of total LCMV L or S segment RNA species via standard or QRT-PCR.

Several approaches have been utilized for the purpose of detecting and/or quantitating the different viral RNA species generated during LCMV infection. These approaches have ranged from standard RT-PCR to screen for the presence or absence of a particular viral RNA species [14,40,41,42] to Northern blot [11], *in situ* hybridization [43,44], or RNase protection assay [14] to more accurately determine the quantities of a given target RNA. Limitations of the latter three assays include the requirement for large quantities of RNA and/or radioactive reagents coupled with limited sensitivity and throughput. The QRT-PCR assay we describe overcomes these limitations and therefore is a valuable research tool for the field. Indeed, several groups from both basic and clinical research settings have reported the use of QRT-PCR approaches to quantitate LCMV RNA load [45,46,47,48,49]. However, these assays were not designed to specifically track individual LCMV replicative RNA species. Additionally, for the assay that theoretically does prime for a specific viral RNA [45], nonspecific cDNA synthesis during RT was not taken into account.

Our initial attempts to develop a strand-specific QRT-PCR assay were complicated by a nonspecific-priming issue whereby cDNA products were generated during RT in the absence of an RT primer. Nonspecific-priming is not exclusive to arenaviruses and is a common issue for both positive- and negative-sense ssRNA viruses [28,29,30,31,32,33,34,35]. In each case, nonspecific-priming during RT makes it impossible to accurately quantify an individual RNA species during the subsequent PCR. Across the various ssRNA virus families, several strategies have been used as a means to overcome this complication. One such strategy is tagged PCR [28,31,32,33,34,35,36,37,50,51,52,53], where RT primers contain a specific tag sequence, not related to the viral sequence in question, that is then subsequently targeted in the PCR. Another strategy has been the use of biotinylated RT primers to permit affinity purification of biotinylated target cDNAs prior to PCR [29,30,36,37]. We were able to successfully utilize the latter strategy to isolate cDNAs derived from specific LCMV RNA species for downstream PCR applications.

There are several means by which nonspecific priming may be occurring. First, the different viral RNA species share significant areas of complementarity and may be self-priming to one another during RT. For example, the GPC mRNA, which is complementary to the S segment vcRNA, could act as a primer for cDNA synthesis of the vcRNA template. Similarly, the NP, L and Z mRNA could prime cDNA synthesis of S vRNA, L vRNA, and L vcRNA, respectively. Likewise, the NP and GPC mRNAs are complementary to one another over stretches of their intergenic regions (IGRs) and each molecule could prime cDNA synthesis of the other (the L and Z mRNAs could do the same). Alternatively, or in combination, host RNA or DNA molecules may also have the ability to “nonspecifically” prime cDNA synthesis from viral RNA templates. It is also possible that the viral RNAs themselves are cleaved by either host nucleases and/or the viral NP [54,55] and that the resulting fragments act to prime the remaining intact viral RNAs for cDNA synthesis. Finally, individual viral RNAs may contain secondary structure that permits “self-priming” to occur. While each of these mechanisms is plausible within an infected cell, it is unlikely that they are all happening in the context of cell-free virions. In particular, it is unlikely that viral RNAs packaged into virions are fragmented. Additionally, priming via viral mRNAs is unlikely to account for the full spectrum of nonspecific priming seen as only the Z mRNA is known to be packaged into viral particles in large quantity [11,56].

We conducted a series of PCR reactions to map the primer-independent cDNAs generated from LCMV S segment RNAs during RT. While full-length S segment cDNAs were not detectable, we were able to amplify PCR products that began at either end of the S segment and, in each case, spanned the adjacent gene, the IGR, and went partially into the gene on the far side of the IGR (as shown in Fig 4). If viral mRNAs were the sole cause of nonspecific priming, we would predict that the IGR on the viral RNA species being primed would not be converted to cDNA because the 3’ end of the mRNA primer would contain part or all of the IGR. Therefore, while our data do not rule out that viral mRNAs can act as primers during RT, they do suggest that additional priming mechanism(s) are occurring. This result could be expected for virion-derived RNAs due to their lack of abundant mRNA for three of the four viral genes [11,56]. Alternatively, if LCMV RNAs are fragmented during infection, the small RNA fragments could act to prime intact viral RNAs for cDNA conversion and generate a series of variable-sized cDNAs. If these cDNAs have regions of complementarity to one another, a PCR primer pair may not amplify a single cDNA of the expected size but rather a collection of smaller cDNAs to produce the full sized product. While our mapping experiments provide basic information regarding the extent that the S segment is converted to cDNA, additional studies will need to be performed to fully define i) the precise composition and diversity of cDNAs generated for both the L and S segment and ii) the mechanism(s) by which these cDNAs are formed. If the precise nature of the nonspecific priming was known, a subtractive method could be used to account for nonspecifically-primed species and permit enumeration of the various viral RNA species without the requirement of a purification step. However, regardless of the primer-independent cDNAs that form during RT or the mechanism responsible for their formation, the assay described here circumvents these issues and allows precise quantification of individual viral RNAs.

We used the assay to characterize the viral RNA content in sucrose-banded LCMV virions. The ratio of S vRNA to L vRNA (1.5 ± 0.1) (Fig 7A and Table 5) observed was consistent with previous studies [15]. We also confirmed the previous RNase protection-based finding that S and L segment vcRNAs are packaged into virions [14] and demonstrated that the quantity of each of these vcRNAs is less than the corresponding vRNA in each case (7.0 ± 0.3-fold less S vcRNA versus S vRNA; 3.3 ± 0.4-fold less L vcRNA versus L vRNA). This finding may help to explain why, in virions, the levels of L and S segment vcRNAs detected in the absence of an RT primer sometimes approached, or slightly exceeded, the quantities seen with the vcRNA-specific RT primer (Fig 3 and Table 4). For instance, it is possible that the quantity of nonspecifically-primed cDNAs generated from the more abundant vRNA templates is close to, or may exceed, the total quantity of vcRNAs available for primer-specific cDNA conversion. Because these vRNA-derived cDNAs can theoretically be detected using the vcRNA QPCR primer-probe set, their presence would increase the copies of vcRNA detected without an RT primer, making this value closer to the quantity seen following the addition of the vcRNA-specific primer. In the case of the cell-free supernatants derived from infected cells at 12 hr, 18 hr, or 24 d pi, the percentage of S segment vcRNA detected without an RT primer was low compared to the values seen with the primer (Fig 3B and Table 4). Because we saw the opposite result (more vcRNA copies without the RT primer than with the RT primer) for the sucrose-banded virions collected at 48 hr pi, this may indicate that the vcRNA content in virions can vary over the course of infection. Alternatively, because the 12 hr, 18 hr, and 24 d pi supernatants were not purified via density ultracentrifugation, is possible that dead or dying cells released S segment RNA species into the media, which could have increased the quantity of vcRNA detected in these samples.

We put considerable effort into optimizing the strand-specific QRT-PCR assay so that it could be of use to researchers in the field for a range of studies. Accordingly, in addition to optimizing the primer, probe, and bead conditions, we also determined the assay conditions required to permit detection of viral RNAs from biological samples containing a full spectrum of viral RNA quantities likely to be encountered during either *in vitro* or *in vivo* infections. For example, the assay was succesfully used to determine RNA copy number of vRNAs and vcRNAs from LCMV-infected tissue culture cells collected at acute or persistent time points following infection as well as sucrose-banded virions. This assay is also applicable for measuring LCMV replicative RNA species from human tissues provided the primers and probe are tailored to match the virus strain of the patient. Several such QPCR primer-probe sets currently exist [46,49], although in each case RT primers specific for a particular LCMV isolate would need to be generated.

While we focused heavily on optimizing the QRT-PCR assay for quantitation of individual LCMV RNA species, the reagents assembled are equally amenable to measuring total L or S segment RNA levels. In this scenario, where the goal is simply to measure viral RNA load across samples, there is no longer a need for affinity purification of a biotinylated cDNA. We have successfully used this approach by including a single primer in the RT reaction (e.g. a primer specific to the vRNA of the L or S segment). Alternatively, to gain sensitivity, both the vRNA- and vcRNA-specific primers for a particular segment can be included in the RT reaction. For example, we have used this approach to reliably detect LCMV S segment RNA in the spleens of mice as few as 3 hours following experimental challenge [57]. Another pratical use of this assay is to measure virion attachment to host cells as we have described for JUNV Candid #1 [58]. Thus, eliminating the affinity purification step potentially increases sensistivity while reducing the cost and labor associated with the assay.

In summary, we describe the first PCR-based assay capable of tracking the quantities of individual arenavirus vRNA and vcRNA to generate a measure of viral replication. The assay has high sensitivity and specificity across a large dynamic range. Through its application to a variety of experimental and clinical samples, it will extend the ability of the field to understand the natural history of LCMV replication over acute and persistent phases of infection.
